# Benchtop study comparing leakages across cuffs of three endotracheal tubes

**DOI:** 10.1186/cc12090

**Published:** 2013-03-19

**Authors:** SM Lam, CW Lau, WW Yan

**Affiliations:** 1 Pamela Youde Nethersole Eastern Hospital, Hong Kong

## Introduction

The aim was to compare two novel endotracheal tubes (ETT), Mallinckrodt TaperGuard (TG, tapered polyvinyl chloride (PVC) cuff) and KimVent Microcuff (MC, cylindrical polyurethrane cuff), with conventional Portex (PT, globular PVC cuff) in leakages across cuffs (microaspiration) under simulated clinical situations. It has been shown that globular PVC cuffs protect poorly against leakages due to microchannels formed from infolding of redundant cuff material [1]. We hypothesized that TG and MC better prevent microaspiration, which is a major mechanism of ventilator-associated pneumonia (VAP).

## Methods

Each ETT was inserted into a silicone cylinder of 2 cm wide inclined at 35°. Then 20 ml water was added above the cuff and leakage measured every minute under five different simulated clinical conditions: mechanical ventilation for acute severe asthma (positive end-expiratory pressure (PEEP) 0 cmH_2_O), normal lungs (PEEP 5 cmH_2_O) and acute respiratory distress syndrome (PEEP 10 cmH_2_O), and disconnection from the ventilator with and without spontaneous breathing effort. Spontaneous breathing was simulated with a respiratory gas exchange simulator. Suction was applied at 200 cmH_2_O sustained for 3 minutes at the Murphy eye. Each scenario was repeated with cuff pressures (Pcuff) 10, 20 and 30 cmH_2_O maintained by a Pcuff maintenance device.

## Results

**Figure 1:**
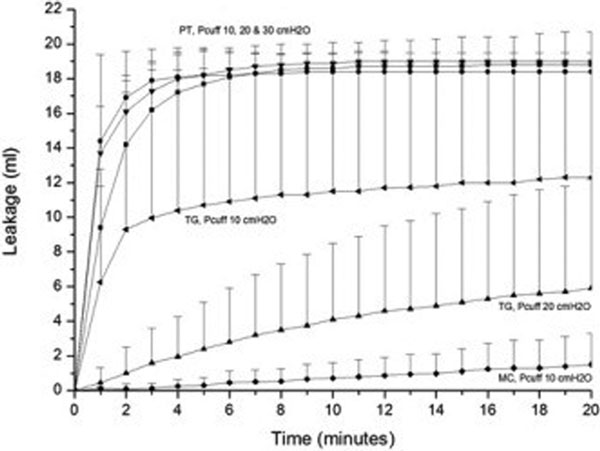
**Downward leak in PEEP 0 cmH_2_O without suction**. Each point represents the mean and standard deviation of eight repeated measures.

PT leaked grossly in all scenarios without PEEP and at PEEP 5 cmH_2_O in the presence of suction irrespective of Pcuff (Pcuff 30 cmH_2_O: PT 19.7 ± 0.4, TG 0.0 ± 0.1, MC 0.0 ± 0.0, *P <*0.001; Pcuff 20 cmH_2_O: PT 19.9 ± 0.4, TG 7.4 ± 6.2, MC 0.0 ± 0.0, *P <*0.001; Pcuff 10 cmH_2_O: PT 20.0 ± 0.0, TG 12.7 ± 5.1, MC 0.9 ± 0.8, *P <*0.001). Leakage under these scenarios can be reduced in TG and prevented in MC by Pcuff ≥20 cmH_2_O (Figure 1).

## Conclusion

Microcuff outperformed the others in preventing microaspiration, while Portex leaked grossly even at a recommended Pcuff of 20 to 30 cmH_2_O whenever PEEP was lost. The effect of ETT type on the incidence of VAP warrants further investigation.
